# Using fuzzy string matching for automated assessment of listener transcripts in speech intelligibility studies

**DOI:** 10.3758/s13428-021-01542-4

**Published:** 2021-03-10

**Authors:** Hans Rutger Bosker

**Affiliations:** 1 Max Planck Institute for Psycholinguistics, PO Box 310, 6500 AH Nijmegen, The Netherlands; 2 Donders Institute for Brain, Cognition and Behaviour, Radboud University, Nijmegen, The Netherlands

**Keywords:** Speech intelligibility, Fuzzy string matching, Transcription accuracy, Automated assessment, Token sort ratio

## Abstract

Many studies of speech perception assess the intelligibility of spoken sentence stimuli by means of transcription tasks (‘type out what you hear’). The intelligibility of a given stimulus is then often expressed in terms of percentage of words correctly reported from the target sentence. Yet scoring the participants’ raw responses for words correctly identified from the target sentence is a time-consuming task, and hence resource-intensive. Moreover, there is no consensus among speech scientists about what specific protocol to use for the human scoring, limiting the reliability of human scores. The present paper evaluates various forms of fuzzy string matching between participants’ responses and target sentences, as automated metrics of listener transcript accuracy. We demonstrate that one particular metric, the token sort ratio, is a consistent, highly efficient, and accurate metric for automated assessment of listener transcripts, as evidenced by high correlations with human-generated scores (best correlation: *r =* 0.940) and a strong relationship to acoustic markers of speech intelligibility. Thus, fuzzy string matching provides a practical tool for assessment of listener transcript accuracy in large-scale speech intelligibility studies. See https://tokensortratio.netlify.app for an online implementation.

## INTRODUCTION

Many studies of speech perception are concerned with the processing of speech in adverse listening conditions. How successful are human listeners in perceiving speech when there is a competing talker in the background (‘cocktail party’ listening), when there is loud noise masking the target speech signal (speech-in-noise), or when the speech signal itself is degraded (e.g., noise-vocoded, dysarthric, accented speech)? To investigate these questions, researchers often ask human listeners to report the lexical content of manipulated or degraded spoken stimuli by means of orthographic transcription (‘type out what you hear’). The intelligibility of the auditory stimulus is then typically assessed by scoring the percentage of words correctly reported from the target stimulus (henceforth: percentage words correct; PWC), following a particular scoring protocol.

The manual scoring of participants’ responses in terms of PWC is a valuable index of intelligibility, demonstrating for instance a positive linear relationship with the BOLD signal in fMRI in classic ‘language’ areas in bilateral temporal cortices (Erb et al., 2013). Furthermore, human scorers are flexible and can score the raw responses while taking into account potential semantic, syntactic, or orthographic constraints motivated by the particular study design (e.g., counting non-literal responses containing synonyms, conjugated forms, or obvious spelling errors as correct). However, this flexibility also presents concerns: there is for instance no consensus on what protocol to follow when scoring PWC. Should scorers assess the accuracy of all words in the target sentence, or only the content words, or even just a few preselected keywords? How should obvious (or less obvious) spelling errors be scored, and what about conjugated forms of the words in the target sentence (e.g., transcribing ‘work’ instead of ‘worked’)? This lack of consensus means that a given response may receive different PWC scores from different human scorers, or even from the same scorer at different occasions. Another drawback of manually scoring PWC is that it is a time-consuming and resource-intensive task. A recent estimate of the time one human scorer spends on scoring PWC is 2.86 seconds per word in the response (Borrie et al., 2019); extrapolating this estimate to a typical study design with 24 participants, 100 sentences, and 10 words per sentence leads to an estimate of more than 19 hours of human scoring time. And this estimate does not yet include the time a second scorer would need to allow for inter-rater reliability checks.

Automated assessment of participants’ accuracy in speech intelligibility tasks could therefore potentially provide an efficient and reliable alternative to human-generated PWC scores. Besides some in-house-developed algorithms (Allison & Hustad, 2014; Wild et al., 2018), there is one recently published application for automatic assessment of listener transcripts: Autoscore (Borrie et al., 2019). This tool, at the most basic level, counts words in transcripts as correct if they match the words in the target phrase exactly, regardless of word order. However, one of the limitations of Autoscore is its sensitivity to spelling errors: non-literal transcripts are scored as incorrect regardless of the degree of orthographic match. For instance, the misspelling ‘wayer’ for the word ‘water’ (e.g., resulting from accidentally pressing [Y] instead [T] on a regular keyboard) will be scored as completely incorrect by Autoscore, despite its orthographic similarity. Autoscore does provide methods to ‘pre-process’ input data, allowing it to deal with grammatical variation (e.g., a plural form in the participant’s transcription, while there was a singular in the target stimulus), but these methods are specifically tailored to English syntax. Autoscore can in principle also deal with misspellings, counting misspellings of target words as correct if they occur in a preloaded default acceptable spelling list including approximately 300 common acceptable spellings. Still, this default list only works for English material. Moreover, it is impossible to know a priori exactly which misspellings will occur in a particular study design, limiting the power of these ‘pre-processing’ tools.

This paper evaluates the consistency, efficiency, and accuracy of various forms of *fuzzy string matching* as automated metrics of participants’ accuracy in speech intelligibility tasks. Fuzzy string matching (also known as ‘approximate string matching’) is a technique to find strings that match a target string approximately, rather than exactly (for an overview, see Singla & Garg, 2012). Common applications are found in record linkage (Wang et al., 2011), spelling checkers, spam filters (Wei et al., 2009), and also speech recognition (Schalk & Zimmerman, 2005; Wu & Chen, 2001) and acoustic model training (Madan et al., 2020). However, it has not yet been applied to transcript accuracy assessment. Fuzzy string matching can be performed using string edit distance (e.g., Levenshtein distance), and more efficient algorithms have been developed based on dynamic programming (Wei et al., 2009). What all computational implementations of fuzzy string matching share is that they quantify the match between a given string and a target string based on the number of shared characters. It is exactly this quantification property that may prove useful for many speech intelligibility studies, where researchers typically seek to quantify the accuracy of a particular transcription given the target sentence.

The present study compares the suitability of three types of fuzzy string matching algorithms, namely the Levenshtein distance (LS), the Jaro distance (J), and the token sort ratio (TSR). Of course, many other possible fuzzy metrics have been developed (e.g., *q*-gram distance, Jaccard distance, cosine distance, etc.; see for example the range of methods of the stringdist() function in the stringdist package in R). The current selection presents a set of metrics that range from commonly known to rather unfamiliar, from generic to more applied, and from simple to more complex (see Methods for detailed motivation of the selected metrics). Outcomes demonstrate that the token sort ratio shows the highest correlation with human-generated PWC scores across six experiments with different experimental designs (‘cocktail party’ listening and speech-in-noise) and the best relation to acoustic markers of speech intelligibility.

## METHODS

### Fuzzy metrics

#### Levenshtein distance (LS)

The first fuzzy string matching metric is the Levenshtein distance (LS; Levenshtein, 1966). This metric was included since it is widely known, forms the core of many other types of fuzzy string matching, is very intuitive, and has been used before in speech intelligibility studies (e.g., Sohoglu & Davis, 2016). The LS metric counts the number of deletions, insertions, and substitutions required to convert the response string into the target string. For instance, say a participant performing a transcription task is presented with a recording of the word ‘water’, but types out ‘wayer’ with a typo (accidentally hitting [Y] instead of [T]). Then, *target =* "water", *response =* "wayer", and to convert *response* into *target* requires one substitution; hence this yields a Levenshtein distance of 1 (see row A2 in Table 1). The Levenshtein distance can easily grow to larger values when working with sentence stimuli; for instance, with *target =* "The big blue house is for sale", and *response =* "sail" (perhaps because the spoken sentence was presented in noise), the Levenshtein distance is 27 (see row B3 in Table 1). That is, one would need to add "The big blue house is for" (25 characters), remove the "i" (1 character), and add a final "e" (1 character) to convert the response string into the target string. Note that a value of 0 indicates a perfect match, and the greater the mismatch, the higher the value. As such, we predict a *negative* correlation between Levenshtein distance and human scores of PWC (the greater the Levenshtein distance, the lower the PWC). For the present study, this metric was obtained using R’s native adist() function.

**Table 1:**
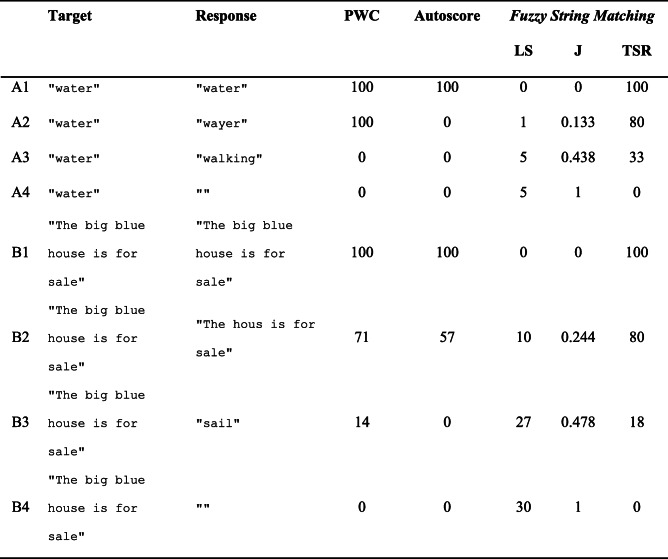
Examples of various metrics of participants’ accuracy in speech intelligibility tasks

PWC = percentage words correct; LS = Levenshtein distance; J = Jaro distance; TSR = token sort ratio. PWC is calculated as a percentage (number of shared words divided by total number of words in target, multiplied by 100), allowing for misspellings. Autoscore values are given in percentages using the default settings

#### Jaro distance (J)

The Jaro distance is a heuristic distance measure and was included in the present study since it was specifically developed for matching human-typed orthographic records with inaccuracies introduced through typing. It was first introduced by the U.S. Bureau of the Census as a practical tool for linking records based on relatively short inaccurate text fields (e.g., name and address strings; Jaro, 1980). With this application in mind, the Jaro distance assumes that typing errors underlie character mismatches and transpositions, yet matches between remote characters are unlikely to have been caused by a typing error (Van der Loo, 2014).

The Jaro distance is calculated by the function:

1 $$ 1-\left(\frac{1}{3}\right)\ast \left(\frac{s}{A}+\frac{s}{B}+\frac{s-n}{s}\right) $$

where *A* = length of *target*; *B* = length of *response*; *s* = number of matching characters between *target* and *response*; *n =* number of necessary transpositions of shared characters. Note that the value of *s* is subject to a cut-off threshold for the distance between two matching characters. That is, two identical characters in the target and response strings are only considered to match if they are no further apart than the length of the longest input string, divided by 2, minus 1 (calculated based on character positions in their respective strings, counting from the left).

For instance, in our example with *target =* "water", *response =* "wayer": *A* = 5; *B* = 5; *s* = 4; *n =* 0, yielding a Jaro distance of 0.133 (see row A2 in Table 1). The calculation of the Jaro distance for longer sentence strings is more complex. For row B3 in Table 1, *target =* "The big blue house is for sale", *response =* "sail", *A* = 30; *B* = 4, *n* = 0. The value of *s* is dependent on the threshold for matching characters, which is set at (30/2) − 1 = 14. As a result, the "i" in "big" (character position 6) and the "l" in "blue" (character position 10) in the target are considered to match the "i" (character position 3) and "l" (character position 4) in the response. However, the "sa" substring in the target does *not* match the "sa" substring in the response because the distance in character positions exceeds the threshold of 14. Hence, *s* is set at 2, resulting in a Jaro distance of 0.478 (see Table 1). This example thus illustrates that the Jaro distance is specifically tailored to relatively short input strings.

Identical strings yield a Jaro distance of 0, strings without matching characters yield 1 (see Table 1). Thus, we predict a *negative* correlation between Jaro distance and human scores of PWC (the greater the Jaro distance, the lower the PWC). This metric was obtained using the function stringdist(method = "jw") in the package stringdist in R (Van der Loo, 2014). Because the output of this function gives the Jaro distance in ratios from 0 to 1 (i.e., not percentages from 0 to 100), we use the same scale here. Note that the Jaro distance is equal to the Jaro–Winkler distance with *p =* 0 (Van der Loo, 2014).

#### Token sort ratio (TSR)

The token sort ratio is a method built into the Python module fuzzywuzzy, created by Adam Cohen at SeatGeek (https://github.com/seatgeek/fuzzywuzzy). This metric was included in the present study because it has been specifically developed for fuzzy string matching of longer string sequences containing multiple words, such as sentences. Also, researchers are already starting to discover its potential for automatic transcription accuracy assessment (Kaufeld et al., 2020).

The fuzzywuzzy module builds on the SequenceMatcher class from the Python module difflib (https://github.com/python/cpython/blob/master/Lib/difflib.py). The function token_sort_ratio() takes two strings as input and returns a measure of the similarity of the two strings between 0 (no match) and 100 (complete match). Because the output of this function is on the percentage scale from 0 to 100 (i.e., not ratios from 0 to 1), we use the same scale here.

The TSR is calculated by first sorting the constituent tokens (i.e., words) of each input string alphabetically (hence the name of the metric; naturally, this only affects sentence strings, not isolated words), and then calling SequenceMatcher to calculate the ratio between the two internally alphabetically ordered input strings. This ratio is calculated as:

2 $$ 2\ast \frac{M}{T}\ast 100 $$

where *M* = the sum of the length of all substrings shared between *target* and *response*, and *T =* the sum of the length of *target* and *response*. In our example with *target =* "water", *response =* "wayer", there are two shared substrings ("wa" and "er") with a summed length of 4. Hence, *M* = 4; *T* = 10, yielding a TSR of 80. For our example in row B2 in Table 1, the TSR is given by first sorting the words inside the sentence strings alphabetically (*target =* "big blue for house is sale the"; *response =* "for hous is sale the"). The ratio between these two ordered strings is then calculated by *M* = 16 (i.e., matching substrings are "for hous" and "is sale the"; not counting whitespace); *T* = 40 (not counting whitespace), yielding a TSR of 80. Identical strings have a TSR of 100, strings without any matching characters have TSR = 0, as does any empty string response (see Table 1). Thus, we predict a *positive* correlation between TSR and human scores of PWC (the greater the TSR, the higher the PWC).

### Data sets

#### Data set A: ‘Cocktail party’ listening

This data set includes sentence transcription data from five experiments (total *N* observations = 2558 from 128 participants), reported in Bosker, Sjerps, and Reinisch (2020b; Experiments 3, 4, 5) and Bosker, Sjerps, and Reinisch (2020a; Experiments 2a and 2b). In all these experiments, Dutch sentences produced by two female native speakers of Dutch were dichotically presented (i.e., one in each ear) to participants over headphones with instructions to attend to only one of the talkers and ignore the other (signal-to-noise ratio (SNR) = 0 dB). The material involved semantically ordinary Dutch sentences, ranging from 11 to 26 syllables, and from 5 to 20 words in length. Although not particularly relevant for our present purposes, note that sentences were manipulated in their speech rate (Bosker et al., 2020b) or first formant frequency (Bosker et al., 2020a), affecting the perception of a sentence-final target word. After first categorizing the sentence-final target word, participants were instructed to type out the sentence produced by the attended talker. Participants were instructed to type out as many words from the attended sentence as possible and to guess if necessary. Responses were scored by human scorers in terms of PWC by dividing the number of content words reported from the target sentence by the total number of content words in the target sentence.

#### Data set B: Speech-in-noise

This data set includes sentence transcription data from a speech-in-noise perception experiment reported in Bosker and Cooke (2020; Experiment 2). In this experiment, 35 listeners—all native speakers of Dutch—were presented with read Dutch sentences elicited from 42 talkers (total *N* observations = 3756). The material involved sentences from the folk tale “The tortoise and the setting sun”, with sentence length ranging from 4 to 47 syllables, and from 5 to 33 words. These sentences were presented diotically over headphones mixed with speech-shaped noise (SSN) at an SNR of −5 dB. Critically, half of the sentences had been produced by the talkers in quiet—referred to as ‘plain speech’—while the other half had been produced by the talkers while hearing loud SSN over headphones at 85 dB A-weighted sound pressure level (SPL)—referred to as ‘Lombard speech’. Based on the listeners’ PWC scores (manually scored counting all words in the target sentence), Bosker and Cooke (2020) report that Lombard speech is more intelligible than plain speech, even when presented at the same SNR; an effect known as the Lombard intelligibility benefit in noise. Moreover, Bosker and Cooke describe a particular acoustic marker of speech intelligibility in noise, namely the power of amplitude modulations in the 1–8 Hz range (cf. Bosker & Cooke, 2018). They show that those talkers that produced more pronounced amplitude modulations in the 1–8 Hz range were observed to be more intelligible than talkers with lower power in that modulation range. Thus, they conclude that enhanced amplitude modulations contribute to the intelligibility benefit of Lombard speech.

## RESULTS

Before any of the metrics were calculated, all target and response strings were ‘pre-processed’ by automatically removing punctuation and excessive whitespace, and by converting all characters to lowercase.

### Consistency and efficiency

Considering that all automated metrics evaluated here are grounded in mathematical functions, their consistency in terms of ‘replicability’ is perfect. They are also extremely efficient compared to generating human PWC scores: on a computer system with a 1.8 GHz Intel Core i7 processor and 16 GB of RAM, calculating all scores for all data sets in one go in R (LS and J) and Python (TSR) took under 30 seconds. This is comparable to the reliability and efficiency of Autoscore (Borrie et al., 2019). However, following the estimate of 2.86 seconds per transcribed word for generating PWC scores (Borrie et al., 2019), human scorers would be estimated to take over 40 hours to score the entire data set of 51,987 words.

### Accuracy

#### TSR shows highest correlations with human-generated PWC scores

We calculated correlations (Pearson’s *r*) between each automated fuzzy string-matching metric and the human PWC scores (see Table 2), separately for each data set, and also across all available data. The correlations go in the expected direction: negative for LS and J; positive for Autoscore and TSR. The strongest correlations to human-generated PWC scores were observed for TSR (overall *r =* 0.922), with a maximum of *r =* 0.940 in the speech-in-noise data set B.

**Table 2:** Correlations (Pearson’s *r*) of automated metrics of listener transcripts with human PWC scores. PWC = percentage words correct; LS = Levenshtein distance; J = Jaro distance; TSR = token sort ratio

Dataset	LS ~ PWC	J ~ PWC	TSR ~ PWC	Autoscore ~ PWC
A: ‘cocktail party’ listening	−0.803	−0.808	0.893	0.854
B: speech-in-noise	−0.790	−0.808	0.940	0.929
Overall	−0.790	−0.803	0.922	0.898

#### TSR shows best relation to acoustic predictors of intelligibility

In the original study about the speech-in-noise data set B (Bosker & Cooke, 2020; Experiment 2), the authors reported that speech produced in noise (Lombard speech) was more intelligible in noise—measured in human PWC scores—when compared to speech produced in quiet (plain speech), even when played at the same SNR. This phenomenon, known as the Lombard speech intelligibility benefit in noise, was first discovered by Dreher and O’Neill (1957) and has been replicated in a large body of literature ever since (Chung et al., 2005; Junqua, 1993; Lu & Cooke, 2008; Pittman & Wiley, 2001; Summers et al., 1988). Given this robust finding, we argued that in principle, indices of transcript accuracy should reflect this acoustic effect. That is, the various indices of transcript accuracy for a given spoken stimulus should correlate with the ‘speech type’ (Lombard speech vs. plain speech) of that stimulus, with better scores for Lombard speech stimuli compared to plain speech stimuli. As such, the index should be able to ‘predict’ the speech type of the target stimulus that formed the basis of the transcription. Metrics that perform worse at predicting the speech type of the stimulus hence are considered to reflect the intelligibility of the stimulus to a lesser degree. Conversely, the metric that predicts the speech type the best may be considered to be the best predictor of stimulus intelligibility.

Therefore, we quantified how well the various automated metrics of listener transcript accuracy could predict the Lombard speech intelligibility benefit. One generalized linear mixed model with a logistic linking function (Quené & Van den Bergh, 2008) was built for each automated metric, attempting to predict the speech type of each individual trial in the speech-in-noise data set B by one automated metric of listener transcript accuracy. Hence, the dependent variable was the binomial variable Speech Type, with plain speech coded as 0, and Lombard speech as 1. The automated metrics were *z*-scored before entering them into the models to improve model fitting. We included random intercepts for Talker and Listener, with by-Talker and by-Listener random slopes for the *z*-scored automated metrics; see Eq. 3 in lme4 syntax.


3

Additionally, we built a ‘null model’ with the same structure as Eq. 3 except that no fixed effects were included. The fit of these models to the observed data was assessed by comparing the various models to the null model using log-likelihood ratio tests with the anova() function in lme4. Note that a log-likelihood ratio test on models M1 and M2 is only allowed if model M1 is nested in model M2 (Baayen, 2008). Hence, we could statistically compare the models with an automated metric as fixed effect to the null model, but statistical comparisons among different models with different automated metrics as fixed effects were not feasible (i.e., only observational numerical comparisons are reported).

All models showed significant improvement in model fit relative to the null model (see Table 3), yet the model with TSR as the predictor showed the numerically best fit to the data as indexed by the log-likelihood closest to zero and the highest *χ*^2^ coefficient.

**Table 3:** Model comparisons for predicting speech type (plain vs. Lombard). PWC = percentage words correct; LS = Levenshtein distance; J = Jaro distance; TSR = token sort ratio; *df* = degrees of freedom. Log-likelihood values closer to zero demonstrate better fit to the data

Model comparison	Log-likelihood	*χ*^2^	*df*	*p*
Null model	−2603.5			
PWC vs. null model	−2399.6	407.74	5	< 0.001
LS vs. null model	−2495.6	215.69	5	< 0.001
J vs. null model	−2446.4	314.04	5	< 0.001
TSR vs. null model	−2386.7	433.51	5	< 0.001
Autoscore vs. null model	−2400.2	406.46	5	< 0.001

Bosker and Cooke (2020) also reported that individual talkers’ overall intelligibility (measured by human PWC scores) correlated with an acoustic property of the speech of those talkers, namely the normalized power of amplitude modulations in the 1–8 Hz range. Therefore, a similar approach as above was applied to assess the relationship between the various automated metrics of listener transcript accuracy and the aforementioned acoustic correlate of intelligibility. Once again, we built a set of linear mixed models (one for each automated metric) that predicted for each individual observation the average normalized power of amplitude modulations in the 1–8 Hz range for that particular talker. The continuous dependent variable and all automated metrics were *z*-scored before entering them into the models to improve model fitting. We included random intercepts for Talker and Listener, with by-Talker and by-Listener random slopes for the *z*-scored automated metrics; see Eq. 4 in lme4 syntax.4

Additionally, we built a ‘null model’ with the same structure as Eq. 4 except that no fixed effects were included. Model fit was assessed by comparing the various models to the null model using the anova() function in lme4. All models showed significant improvement in model fit relative to the null model (see Table 4), yet the model with TSR as the predictor showed the numerically best fit to the data as indexed by the log-likelihood closest to zero and the highest *χ*^2^ coefficient.

**Table 4:** Model comparisons for predicting individual talkers’ normalized amplitude modulation power. PWC = percentage words correct; LS = Levenshtein distance; J = Jaro distance; TSR = token sort ratio; *df* = degrees of freedom. Log-likelihood values closer to zero demonstrate better fit to the data

Model comparison	Log-likelihood	*χ*^2^	*df*	*p*
Null model	−4441.4			
PWC vs. null model	−4166.9	549.04	5	< 0.001
LS vs. null model	−4307.4	267.98	5	< 0.001
J vs. null model	−4252.3	378.24	5	< 0.001
TSR vs. null model	−4142.2	598.55	5	< 0.001
Autoscore vs. null model	−4180.2	522.50	5	< 0.001

## DISCUSSION

This study assessed the suitability of fuzzy string matching as a method for scoring listener transcripts in speech intelligibility research. We compared the consistency, efficiency, and accuracy of three types of fuzzy string matching: Levenshtein distance (LS), Jaro distance (J), and the token sort ratio (TSR). Moreover, we compared each of these to a recently introduced automatic tool for assessment of listener transcript accuracy, namely Autoscore (Borrie et al., 2019), which does not use fuzzy string matching. Instead, it counts words in transcripts as correct if they match the words in the target phrase exactly.

Regarding consistency, all metrics involved mathematical functions and hence their reliability in terms of replicability is perfect. This is an important improvement compared to human-generated scores of percentage words correct (PWC) which may vary between and within human scorers, particularly when the scoring protocol is complex (i.e., when having to take into account multiple exceptions related to misspellings, grammatical variation, synonyms, etc.). Another advantage of the use of mathematical algorithms is that they prevent potential human biases entering into the scoring of transcription accuracy. Finally, all automated metrics were also highly efficient, performing the scoring of thousands of observations in a matter of seconds. This is also an improvement compared to human PWC scores which typically take hours of manual labor to generate for a data set of reasonable size.

To assess accuracy, we assessed the relationship between the various automated metrics and human PWC scores. Across multiple data sets, including various experimental designs and tasks, we observed the strongest correlation between human PWC scores and TSR (overall *r =* 0.922). The TSR also demonstrated the strongest relationship to two acoustic markers of intelligibility, namely the Lombard intelligibility benefit in noise and individual talkers’ power of amplitude modulations in the 1–8 Hz range. Thus, the present investigation suggests that TSR is a consistent, efficient, and accurate method for accuracy assessment of listener transcripts in speech intelligibility studies.

We may speculate as to the reasons why TSR outperformed the other automated metrics. LS is presumably a rather basic form of fuzzy string matching, since the LS scale is unbounded (i.e., an empty response receives its LS score depending on the length of the target string). As a result, a data point with zero PWC score can have any LS score, reducing their correlation. Note that normalizing LS by dividing it by the length of the target string does not wholly handle this concern. That is, even such normalized LS scores are unbounded since response strings that are longer than the target string can still take values >1. In contrast, J is bounded between 0 and 1, improving the correlation between J and PWC scores. Still, J has been designed for relatively short string sequences (e.g., name and address), penalizing data points where the target is a sentence, but the response is only one or two words from that sentence (see example B3 in Table 1 in Methods). Since sentences are typically used as stimulus materials in many speech intelligibility studies, this reduces the suitability of J for automated assessment of listener transcripts. Autoscore demonstrated high correlations with human PWC scores. Still, it is sensitive to misspellings, counting non-literal response words as entirely incorrect despite any potential orthographic match (e.g., response ‘wayer’ for target ‘water’). By contrast, TSR returns a measure of the orthographic similarity of the response to the target string, making it less easily affected by misspellings.

At the same time, this strength of TSR also has its shortcomings. For instance, entirely inaccurate responses that happen to share characters with the target string still receive a TSR score greater than zero. This property of TSR means that it tends to overestimate the intelligibility of stimuli with a relatively low PWC (i.e., below 25). However, distinguishing misspellings from errors is not straightforward, and this concern applies to all measures of listener transcript accuracy, be they human-generated or automatically calculated. For instance, is the response ‘waiter’ for target ‘water’ an error (the participant erroneously reported ‘waiter’) or a misspelling (the participant aimed to report ‘water’ but made a typo)? Another characteristic of the TSR is that it sorts the words in sentence strings alphabetically before performing its calculations (hence its name). This means that word order is disregarded in assessing transcript accuracy; for instance, the response ‘John saw Mary’ for the target sentence ‘Mary saw John’ would receive a perfect score of 100. Still, we should point out that this concern is not specific to the TSR; all automated metrics and many human PWC scoring protocols ignore word order. Therefore, this issue invites further debate on whether to take word order into consideration when assessing transcript accuracy in speech intelligibility studies, and if so how to quantify word order variability. Finally, the TSR—like the other automated metrics presented here—quantifies the orthographic match between target and response strings, not the phonological match. This may be striking considering that they are applied to transcription data resulting from speech listening. As a result, the response ‘eye’ for the target pronoun ‘I’ is considered an inaccurate response with a low score, despite the perfect phonological match. Still, it also means that the match between response ‘wter’ (with a typo) for target ‘water’ can be quantified, despite the fact that the response string cannot be pronounced in English. Future work may assess whether quantifying the phonological match between target and response strings through the incorporation of a pronunciation dictionary (e.g., using ALINE; Kondrak, 2003; Podlubny et al., 2018) provides a closer approximation of speech intelligibility than the present orthographic metrics do. Nevertheless, one should note that such solutions would necessarily be language-specific, hindering the applicability of the tool to other languages.

Note that the present study explicitly *does not claim* that TSR is always the best measure for any given experimental design, nor does it claim that other measures of listener transcript accuracy are unsuited for speech intelligibility research. The differences between the various metrics evaluated here were small; Autoscore in particular performed well. Stimulus characteristics, experimental design, and data quality all factor into the selection of the appropriate automated metric. If the design uses single-word stimuli, perhaps even testing minimal pairs (e.g., ‘bath’ vs. ‘path’ in a study of voice-onset time), researchers may want to strictly score the transcription accuracy, disregarding any orthographic similarity. In such cases, Autoscore would be more suitable. For smaller data sets, when close precision is required, researchers may opt for human-generated PWC scores instead of automated metrics, despite the reduced efficiency. One reason for this decision in such circumstances could be the flexibility of human scorers, for instance when elaborate scoring protocols are required. However, even for such designs, automated metrics can be highly valuable by allowing one to quickly filter out the ‘perfect’ (TSR = 100) and ‘entirely inaccurate’ (TSR = 0) responses, thus reducing the scoring load on human scorers. Still, the present analyses demonstrate that, in particular for large-scale investigations, comparing for instance different listening conditions or forms of speech degradation, with large numbers of listeners and long target strings (i.e., sentence materials), TSR closely approximates human PWC scores with much greater efficiency.

In fact, the accuracy and efficiency of TSR opens up new opportunities in speech intelligibility research. Although only Dutch materials were tested here, TSR scores can be easily and quickly calculated for any language, with any type of orthography, since UTF-8 encoding is supported. It also creates an opportunity for real-time feedback on listener performance during the running of a speech intelligibility experiment, which may prove especially useful for training studies and/or when individualized perceptual thresholds are required (e.g., speech reception thresholds; SRT). Finally, it may also prove useful in clinical settings: assessment of the speech perception skills of individuals with hearing impairments may be facilitated with TSR as an automated metric of transcription accuracy.

## TOOL AVAILABILITY

Researchers may calculate the token sort ratio for their own transcription data in one of two ways:

1. Researchers may upload their own data in .csv format to the online point-and-click interface implementation at https://tokensortratio.netlify.app. This online interface calculates the TSR score on the input columns labelled ‘target’ and ‘response’ with default settings.
2. Alternatively, they may download, perhaps adapt, and then run the Python script provided on the Open Science Framework (OSF): https://osf.io/73dnj/. This script contains the same functions called by the online tool mentioned above, but allows for customizing the settings.

These two implementations are identical in their code and output, taking a data frame as input with minimally two columns: one containing the target sentences (column ‘target’) and one containing the participants’ orthographic responses (column ‘response’). The output is the same input data frame extended with one additional column ‘TSR_score’ containing the token sort ratio score between 0 (zero accuracy) and 100 (perfect accuracy). Both the online tool and the provided Python script on OSF are based on the token_sort_ratio() function in the fuzzywuzzy Python module, created by Adam Cohen at SeatGeek (https://github.com/seatgeek/fuzzywuzzy).
